# Expression of CAF-Related Proteins Is Associated with Histologic Grade of Breast Phyllodes Tumor

**DOI:** 10.1155/2016/4218989

**Published:** 2016-11-01

**Authors:** Hye Min Kim, Yu Kyung Lee, Ja Seung Koo

**Affiliations:** Department of Pathology, Yonsei University College of Medicine, Seoul, Republic of Korea

## Abstract

*Purpose*. The purpose of this study was to investigate the expression of cancer-associated fibroblast- (CAF-) related proteins and the implications in breast phyllodes tumor (PT).* Methods*. Tissue microarrays of 194 PT cases (151 benign PT, 27 borderline PT, and 16 malignant PT) were constructed. We performed immunohistochemical staining for CAF-related proteins (podoplanin, prolyl 4-hydroxylase, FAP*α*, S100A4, PDGFR *α*/*β*, and NG2) and analyzed the results according to clinicopathologic parameters.* Results*. Expression of PDGFR*α* and PDGFR*β* in the stromal component increased with increasing histologic grade of PT (*p* = 0.003 and *p* = 0.034, resp.). Among clinicopathologic parameters, only expression of FAP*α* in stroma was associated with distant metastasis (*p* = 0.002). In univariate analysis, stromal expression of PDGFR*α* was associated with shorter overall survival (*p* = 0.002). In Cox multivariate analysis, stromal overgrowth and PDGFR*α* stromal positivity were associated with shorter overall survival (*p* = 0.006 and *p* = 0.050, resp.). Furthermore, expression of PDGFR*β* in stroma was associated with shorter overall survival in patients with malignant PT (*p* = 0.041).* Conclusion*. Stromal expression of PDGFR*α* and PDGFR*β* increased with increasing histologic grade of PT. In addition, PDGFR stromal positivity was associated with shorter overall survival. These results suggest that CAFs are associated with breast PT progression.

## 1. Introduction

Progress in cancer research has increasingly revealed the clinical significance of the tumor microenvironment. Among various components of the tumor microenvironment, cancer-associated fibroblasts (CAFs), one of the most important elements, have been widely studied [1]. CAFs are located near the cancer cells and have been reported to be involved in tumor initiation, tumor-stimulatory inflammation, metabolism, metastasis, drug response, and immune surveillance [2]. Despite their significant effect on cancer cells, the exact cell origin of CAFs is not completely understood and there is even controversy concerning the definition of CAF [1, 2]. Various proteins have been suggested as markers for CAFs, including *α*-smooth muscle actin (SMA) [3], tenascin-C [4], chondroitin sulfate proteoglycan (NG2) [5], platelet-derived growth factor receptor (PDGFR)*α*/*β* [6], fibroblast activation protein (FAP) [7], podoplanin [8], prolyl 4-hydroxylase [9], and fibroblast-specific protein- (FSP-) 1 [5]. CAFs have been suggested to show various functional subtypes that exhibit different characteristics [10], supporting the hypothesis that CAFs have various phenotypes.

Phyllodes tumor (PT) is a relatively rare biphasic breast tumor that accounts for only 0.3–1.5% of all breast tumors [11]. It is composed of atypical spindle cell stroma and usually benign epithelium [12]. Pathologically, differential diagnosis is difficult because several histologic findings of PT overlap with those of fibroadenoma, which is also a fibroepithelial tumor, and PT shows heterogeneous histologic features within the tumor [11, 13]. In addition, some cases of PT show several clinical features of malignancy such as relapse or distant metastasis [14]. The histologic classification of PT varies according to authors, but PT has been classified as benign, borderline, and malignant according to the World Health Organization classification of tumors of the breast [11]. A higher histologic grade of PT is associated with increased risk of tumor recurrence or distant metastasis, indications of tumor aggressiveness. Furthermore, among malignant PT, 9–36% of cases experience local recurrence, 9–40% of cases progress with metastasis to the lungs, brain, or liver [15], and some cases suffer death due to recurrence and distant metastasis [16, 17]. The fibroblast is an important component of PT [18, 19]; therefore, variations in tumor biology of PT could be accounted for by the phenotype of associated fibroblasts. However, the role of CAF-related proteins in breast PT is poorly understood. The purpose of this study was to investigate the expression of CAF-related proteins and its implications in breast PT.

## 2. Materials and Methods

### 2.1. Patient Selection

Tissue samples from patients who were diagnosed with breast PT from 2000 to 2010 in Severance hospital were selected. All tissues were fixed in 10% buffered formalin and embedded in paraffin. Archival hematoxylin and eosin- (H&E-) stained slides for each case were reviewed by a single pathologist (Ja Seung Koo). The PT histologic grade was based on the World Health Organization classification of tumors of the breast [11]. The histologic grade was evaluated with H&E-stained slides and clinical data including patient age, recurrence, distant metastasis, and patient survival were obtained by reviewing the patients' medical records. The study was approved by the Institutional Review Board of Yonsei University Severance Hospital.

### 2.2. Tissue Microarray

Construction of tissue microarray was performed as previously described. Briefly, a representative area was selected on the H&E-stained slide of the tumor and a corresponding spot was marked on the surface of the paraffin block. Using a biopsy needle, the selected area was punched out and the 5 mm tissue core was placed in a 5 × 6 recipient block. To minimize extraction bias, we extracted two tissue cores for every PT case. Finally, each separate tissue core was assigned a unique tissue microarray location number linked to the database including other clinicopathologic data and was used for immunohistochemical staining.

### 2.3. Immunohistochemistry

The antibodies used for immunohistochemical staining in this study are shown in Table 1. All immunostaining was performed using the constructed tissue microarray. For immunohistochemistry, 5 *μ*m sections were obtained with a microtome, transferred onto adhesive slides, and dried at 62°C for 30 min. After incubation with primary antibodies, immunodetection was performed with biotinylated antimouse immunoglobulin followed by peroxidase-labeled streptavidin using a labeled streptavidin biotin kit with 3,3′-diaminobenzidine chromogen as substrate. For the negative control, the primary antibody incubation step was omitted. Harris hematoxylin was used for tissue counterstaining. All immunohistochemical markers were assessed using light microscopy and evaluation of staining was performed by calculating the proportion of stained cells and immunostaining intensity. The proportion of stained cells was defined as follows: 0, negative; 1, less than 30% positive; and 2, more than 30% positive. The immunostaining intensity was defined as follows: 0, negative; 1, weak; 2, moderate; and 3, strong. The scores for proportion of stained cells and immunostaining intensity were multiplied, and staining was defined as positive when the final score was >1 [20].

### 2.4. Statistical Analysis

Data were analyzed using SPSS for Windows, Version 21.0 (SPSS Inc., Chicago, IL, USA). Continuous variables were compared using two-tailed Students' *t*-test and categorical data were compared using the Chi square test. To evaluate the time to tumor recurrence and compare the survivals between groups, Kaplan-Meier survival curves and the log-rank test were used. Multivariate survival analyses using a Cox's proportional hazard model were performed to characterize the prognostic factors in PT. A two-tailed *p* value < 0.05 was considered statistically significant.

## 3. Results

### 3.1. Basal Characteristics of Phyllodes Tumors

This study included 194 cases of breast PT, including 151 cases of benign PT, 27 cases of borderline PT, and 16 cases of malignant PT. The basal characteristics of the patients are shown in Table 2. Greater patient age and larger tumor size were associated with higher PT histologic grade (*p* = 0.017 and *p* = 0.001, resp.). Higher rate of tumor recurrence and distant metastasis were also associated with higher PT histologic grade (*p* < 0.001). Eight cases with distant metastasis showed lung metastasis.

### 3.2. Expression of CAF-Related Proteins according to Phyllodes Tumor Histologic Grade

**Figure 1:**
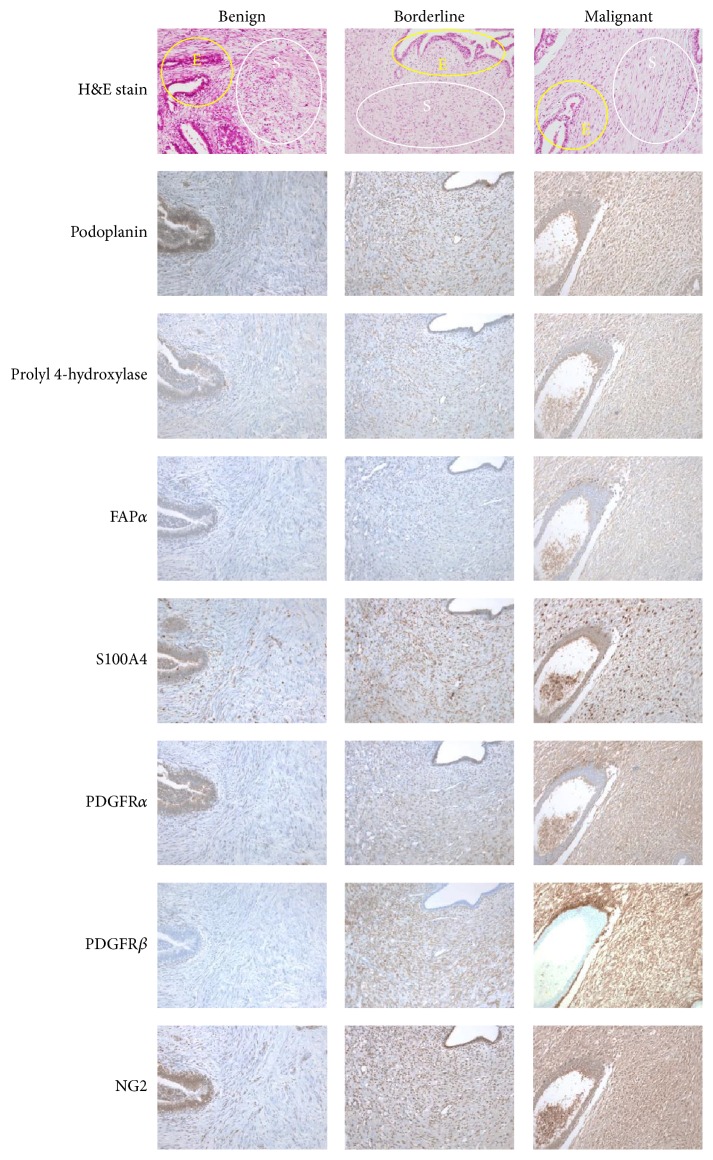
Expression of CAF-related proteins according to the histologic grade of phyllodes tumor. The stromal expression of PDGFR*α* and *β* increased with increasing phyllodes tumor histologic grade. The yellow circle indicates epithelium and the white circle indicates the stroma. CAF, cancer-associated fibroblast; H&E, hematoxylin and eosin stain; FAP, fibroblast activation protein; PDGFR, platelet-derived growth factor receptor; NG2, chondroitin sulfate proteoglycan.

There were no significant differences in immunohistochemical staining in the epithelial component according to PT histologic grade for any of the proteins analyzed (Table 3). For the stromal component, the expression of PDGFR*α* and PDGFR*β* increased with increasing PT histologic grade (*p* = 0.003 and *p* = 0.034, resp.) (Table 4 and Figure 1).

### 3.3. Correlation between the Expression of CAF-Related Proteins in Phyllodes Tumor and Clinicopathologic Parameters

**Figure 2:**
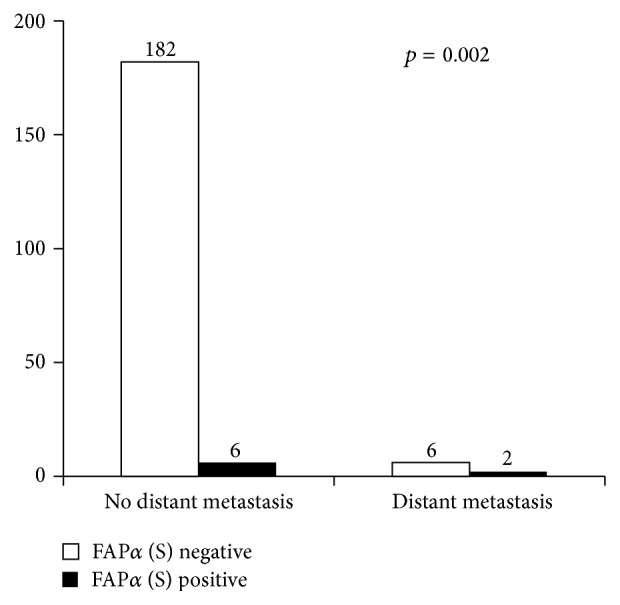
Correlation between stromal expression of fibroblast activation protein (FAP)*α* in phyllodes tumor and distant metastasis.

We investigated the correlation between the expression of CAF-related proteins in PT and clinicopathologic parameters. Only expression of FAP*α* in the stromal cells was associated with distant metastasis (*p* = 0.002) (Figure 2). Other clinicopathologic parameters, including age, tumor size, stromal cellularity, stromal atypia, stromal mitosis, stromal overgrowth, tumor margin, and tumor recurrence, were not associated with the expression of CAF-related proteins in PT.

### 3.4. Impact of Expression of CAF-Related Proteins on Patient Prognosis

**Figure 3:**
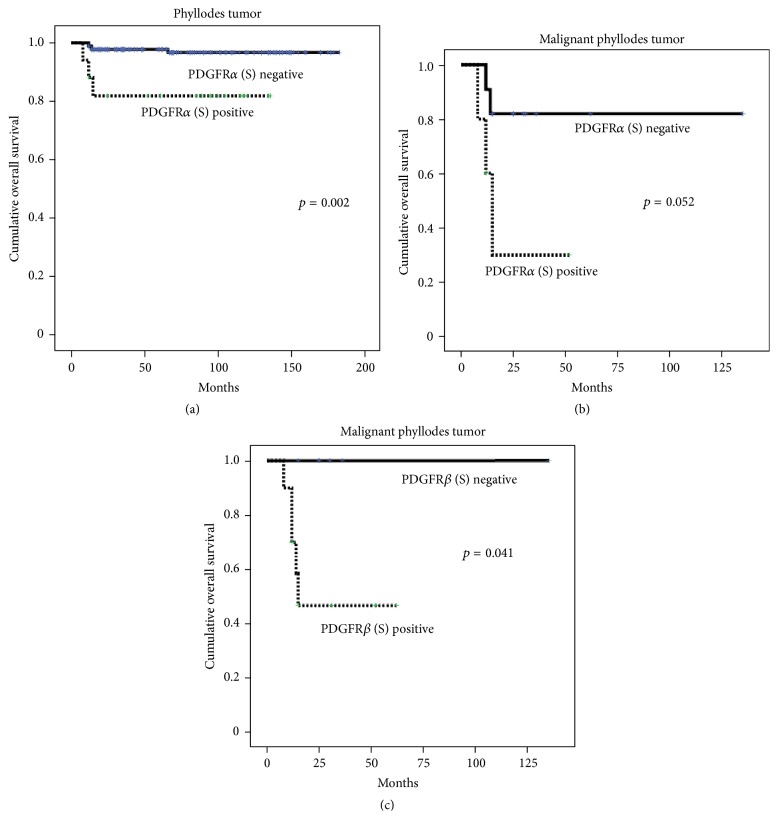
Overall survival curves according to the status of CAF-related proteins in stromal component of phyllodes tumor. (a) Univariate analysis showed that stromal expression of PDGFR*α* was associated with shorter overall survival in phyllodes tumor. (b) Stromal expression of PDGFR*α* was associated with shorter overall survival in malignant phyllodes tumor, although statistical significance was not reached regarding PDGFR*α* expression. (c) Stromal expression of PDGFR*β* was associated with shorter overall survival in malignant phyllodes tumor. CAF, cancer-associated fibroblast; PDGFR, platelet-derived growth factor receptor.

Univariate analysis showed that stromal expression of PDGFR*α* was associated with shorter overall survival (*p* = 0.002) (Table 5 and Figure 3(a)). Furthermore, in Cox multivariate analysis, higher PT histologic grade (hazard ratio: 7.990, 95% CI: 2.196–29.07, and *p* = 0.002) and stromal overgrowth (hazard ratio: 7.288, 95% CI: 1.225–43.35, and *p* = 0.029) were associated with shorter disease-free survival. Regarding overall survival, stromal overgrowth (hazard ratio: 58.10, 95% CI: 3.116–1083, and *p* = 0.006) and PDGFR*α* positivity (hazard ratio: 5.486, 95% CI: 1.003–30.01, and *p* = 0.050) were associated with shorter overall survival (Table 6). Furthermore, stromal expression of PDGFR*α* (*p* = 0.052) (Figure 3(b)) and PDGFR*β* (*p* = 0.041) (Figure 3(c)) was associated with shorter overall survival in malignant PT, although statistical significance was not reached regarding PDGFR*α* expression.

## 4. Discussion

In this study, we investigated the expression of CAF-related proteins in breast PT according to histologic grade with the aim of identifying a new therapeutic target for PT.

Recently, there have been many studies on the tumor microenvironment as a novel therapeutic target. The tumor microenvironment includes nontumor cells with nontransformed elements, including immune system elements (such as macrophages and lymphocytes), blood vessel cells, fibroblasts, myofibroblasts, mesenchymal stem cells, adipocytes, and extracellular matrix, in close proximity to tumor cells. PT is a representative fibroepithelial tumor characterized by fibrous stroma composed of fibroblasts surrounding the epithelium. Previous studies reported that CD34-expressing fibroblasts exist in mammary stroma. Because CD34-expressing fibroblasts were observed in both fibroadenoma and PT [19], efficient treatment for fibroepithelial tumors might be achieved by targeting the fibrous stroma. However, there are no studies on the expression of CAF-related proteins in PT, and as PT shows heterogeneous tumor stromal features, the expression of CAF-related proteins is predicted to vary in each type.

In this study, we performed immunohistochemical staining for CAF-related proteins focusing on the stromal component. Our results showed that the expression of PDGFR*α* and PDGFR*β* increased with increasing PT histologic grade. Platelet-derived growth factor is a major mitogen for several cell types, including connective tissue cells, that is activated by binding to two protein tyrosine kinase receptors (PDGFR*α* and PDGFR*β*). PDGF signaling in tumor cells induces point mutations, amplification, and translocations, which stimulate autocrine stimulatory loops [6, 21]. For example, PDGFR*α* and *β* are two well-known receptors that participate in breast cancer progression [22]. In addition, in breast cancer, the desmoplastic response appears to be mediated by PDGF-AA signaling in PDGFR*α* type CAFs [23]. Desmoplasia refers to the growth of fibrous or connective tissue near the tumor and is increased in tumors with aggressive properties. The results from our study revealed that higher grade PT with more active fibroblasts showed higher expression of PDGFR*α* and *β*, suggesting that fibroblasts with a CAF phenotype are associated with PT progression.

Stromal PDGFR*α* expression was related to the prognosis of patients with PT. Furthermore, subgroup analysis in malignant PT showed that stromal PDGFR*β* expression was associated with shorter overall survival and, although statistical significance was not noted, there was also a trend for increased stromal PDGFR*α* expression. In previous studies of glioma [24], squamous cell carcinoma of the head and neck [25], colorectal cancer [26], pancreatic cancer [27], and T cell lymphoma [28], PDGFR activation induced the intracellular signaling pathway and promoted cell migration, invasion, survival, and proliferation [29]. Similarly, PDGFR activation is related to lymphatic metastasis in pancreatic cancer [30] and gastric cancer [31], suggesting that PDGFR might be used as a prognostic marker in malignant PT. This should be validated in future studies.

We found that expression of FAP*α* in stroma was associated with distant metastasis. Fibroblast activation protein is expressed by reactive CAFs in tumor stroma or granulation tissue and is known to be involved in wound healing. The expression of FAP in CAFs is reported in various carcinomas and is used as an important marker of CAF [32, 33]. In a meta-analysis that analyzed the clinical implication of FAP overexpression in solid tumors of colorectal cancer, pancreatic adenocarcinoma, non-small cell lung cancer, breast cancer, medullary thyroid carcinoma, and oral squamous cell carcinoma, high FAP expression was related to the risk of distant metastasis (OR: 2.56) [34], showing similar results to our study. FAP regulates proteolysis of the extracellular cell matrix in tumor stroma, causing stromal cell proliferation and invasiveness. Therefore, it could be speculated that tumors with high expression of FAP*α* are prone to metastasis to distant organs [35, 36].

The clinical significance of this study is the role of CAFs as a potential therapeutic target. Sunitinib, a US Food and Drug Administration- (FDA-) approved PDGFR inhibitor, is currently used in the treatment of advanced renal cell carcinoma [37], advanced progressive pancreatic neuroendocrine tumor [38], and advanced radioiodine refractory thyroid carcinoma [39]. Furthermore, ongoing preclinical trials are testing the therapeutic effect of another PDGFR inhibitor, imatinib mesylate, in gastrointestinal stromal tumor [40, 41] and* in vitro* and* in vivo* studies of malignant peripheral nerve sheath tumors have shown promising results [42]. Therefore, inhibition of PDGFR in PT might be a potential therapeutic strategy. However, further investigation regarding the effect of these inhibitors in PT is required.

Taken together, our data show that expression of PDGFR*α* and PDGFR*β* in the stromal component increased with increased PT histologic grade. Also, stromal expression of PDGFR was associated with shorter overall survival. Overall, the expression of CAF-related proteins is related to the histologic grade of breast PT and PDGFR inhibitors in particular have potential as novel treatments for PT.

## Figures and Tables

**Table 1:** Source, clone, and dilution of antibodies used for immunohistochemical staining.

Antibody	Company	Clone	Dilution
*CAF phenotype-related proteins*			
Podoplanin	Abcam, Cambridge, UK	18H5	1 : 100
Prolyl 4-hydroxylase	Abcam, Cambridge, UK	Polyclonal	1 : 200
FAP*α*	Abcam, Cambridge, UK	Polyclonal	1 : 100
S100A4	Abcam, Cambridge, UK	Polyclonal	1 : 100
PDGFR*α*	Abcam, Cambridge, UK	Polyclonal	1 : 100
PDGFR*β*	Abcam, Cambridge, UK	Y92	1 : 100
NG2	Abcam, Cambridge, UK	NG2	1 : 50

**Table 2:** Clinicopathologic characteristics of patients with phyllodes tumor.

Parameters	Total *n* = 196 (100%)	PT, benign *n* = 153 (100%)	PT, borderline *n* = 27 (100%)	PT, malignant *n* = 16 (100%)	*p* value
Age (years, mean ± SD)	40.1 ± 12.3	38.9 ± 12.2	42.3 ± 11.5	47.6 ± 12.9	0.017
Tumor size (cm, mean ± SD)	4.0 ± 2.6	3.6 ± 2.1	4.3 ± 2.5	6.7 ± 4.6	<0.001
Stromal cellularity					<0.001
Mild	121 (61.7)	120 (78.4)	1 (3.7)	0 (0.0)	
Moderate	63 (32.1)	33 (21.6)	23 (85.2)	7 (43.8)	
Marked	12 (6.1)	0 (0.0)	3 (11.1)	9 (56.2)	
Stromal atypia					<0.001
Mild	156 (79.6)	151 (98.7)	5 (18.5)	0 (0.0)	
Moderate	30 (15.3)	2 (1.3)	20 (74.1)	8 (50.0)	
Marked	10 (5.1)	0 (0.0)	2 (7.4)	8 (50.0)	
Stromal mitosis					<0.001
0–4/10 HPFs	154 (78.6)	153 (100.0)	1 (3.7)	0 (0.0)	
5–9/10 HPFs	31 (15.8)	0 (0.0)	26 (96.3)	5 (31.2)	
≥10/10 HPFs	11 (5.6)	0 (0.0)	0 (0.0)	11 (68.8)	
Stromal overgrowth					<0.001
Absent	179 (91.3)	153 (100.0)	24 (88.9)	2 (12.5)	
Present	17 (8.7)	0 (0.0)	3 (11.1)	14 (87.5)	
Tumor margin					<0.001
Circumscribed	176 (89.8)	150 (98.0)	20 (74.1)	6 (89.7)	
Infiltrative	20 (10.2)	3 (2.0)	7 (25.9)	10 (62.5)	
Tumor recurrence	18 (9.2)	5 (3.3)	6 (22.2)	7 (43.8)	<0.001
Distant metastasis	8 (4.1)	0 (0.0)	1 (3.7)	7 (43.8)	<0.001

PT, phyllodes tumor; HPF, high-power fields.

**Table 3:** Expression of CAF-related proteins in epithelial component of phyllodes tumor according to histologic grade^*∗*^.

Parameters	Total *n* = 179 (100%)	PT, benign *n* = 151 (100%)	PT, borderline *n* = 23 (100%)	PT, malignant *n* = 5 (100%)	*p* value
Podoplanin					0.329
Negative	35 (19.6)	27 (17.9)	6 (26.1)	2 (40.0)	
Positive	144 (80.4)	124 (82.1)	17 (73.9)	3 (60.0)	
Prolyl 4-hydroxylase					0.621
Negative	174 (97.2)	146 (96.7)	23 (100.0)	5 (100.0)	
Positive	5 (2.8)	5 (3.3)	0 (0.0)	0 (0.0)	
FAP*α*					0.794
Negative	166 (92.7)	140 (92.7)	21 (91.3)	5 (100.0)	
Positive	13 (7.3)	11 (7.3)	2 (8.7)	0 (0.0)	
S100A4					0.571
Negative	93 (52.0)	81 (53.6)	10 (43.5)	2 (40.0)	
Positive	86 (48.0)	70 (46.4)	13 (56.5)	3 (60.0)	
PDGFR*α*					0.738
Negative	145 (81.0)	121 (80.1)	20 (87.0)	4 (80.0)	
Positive	34 (19.0)	30 (19.9)	3 (13.0)	1 (20.0)	
PDGFR*β*					**N/A**
Negative	179 (100.0)	151 (100.0)	23 (100.0)	5 (100.0)	
Positive	0 (0.0)	0 (0.0)	0 (0.0)	0 (0.0)	
NG2					0.216
Negative	60 (33.5)	52 (34.4)	5 (21.7)	3 (60.0)	
Positive	119 (66.5)	99 (65.6)	18 (78.3)	2 (40.0)	

^*∗*^Seventeen tumors without an epithelial component were excluded.

PT, phyllodes tumor.

**Table 4:** Expression of CAF-related proteins in the stromal component of phyllodes tumor according to histologic grade.

Parameters	Total *n* = 196 (100%)	PT, benign *n* = 153 (100%)	PT, borderline *n* = 27 (100%)	PT, malignant *n* = 16 (100%)	*p* value
Podoplanin					0.728
Negative	52 (26.5)	41 (26.8)	8 (29.6)	3 (18.8)	
Positive	144 (73.5)	112 (73.2)	19 (70.4)	13 (81.2)	
Prolyl 4-hydroxylase					0.563
Negative	192 (98.0)	149 (97.4)	27 (100.0)	16 (100.0)	
Positive	4 (2.0)	4 (2.6)	0 (0.0)	0 (0.0)	
FAP*α*					0.105
Negative	188 (95.9)	149 (97.4)	25 (92.6)	14 (87.5)	
Positive	8 (4.1)	4 (2.6)	2 (7.4)	2 (12.5)	
S100A4					0.104
Negative	65 (33.2)	56 (36.6)	7 (25.9)	2 (12.5)	
Positive	131 (66.8)	97 (63.4)	20 (74.1)	14 (87.5)	
PDGFR*α*					**0.003**
Negative	179 (91.3)	142 (92.8)	26 (96.3)	11 (68.8)	
Positive	17 (8.7)	11 (7.2)	1 (3.7)	5 (31.2)	
PDGFR*β*					**0.034**
Negative	80 (40.8)	69 (45.1)	5 (18.5)	6 (37.5)	
Positive	116 (59.2)	84 (54.9)	22 (81.5)	10 (62.5)	
NG2					0.695
Negative	93 (47.4)	75 (49.0)	11 (40.7)	7 (43.8)	
Positive	103 (52.6)	78 (51.0)	16 (59.3)	9 (56.2)	

PT, phyllodes tumor.

**Table 5:** Univariate analysis of the impact of CAF-related proteins in the stromal component of phyllodes tumor on patient prognosis using the log-rank test.

Parameters	Number of patients Total/recurrence/metastasis	Disease-free survival		Overall survival	
		Median survival (95% CI) months	*p* value	Median survival (95% CI) months	*p* value
Podoplanin			0.936		0.494
Negative	52/5/3	160 (146–174)		166 (155–178)	
Positive	144/13/5	166 (158–175)		177 (171–182)	
Prolyl 4-hydroxylase			n/a		n/a
Negative	192/18/8	n/a		n/a	
Positive	4/0/0	n/a		n/a	
FAP*α*			0.099		0.233
Negative	188/16/7	164 (157–171)		172 (167–177)	
Positive	8/2/1	131 (71–191)		162 (123–200)	
S100A4			0.607		0.792
Negative	65/5/3	163 (152–174)		168 (159–177)	
Positive	131/13/5	165 (155–174)		176 (170–182)	
PDGFR*α*			0.698		**0.002**
Negative	179/16/5	167 (159–174)		177 (173–182)	
Positive	17/2/3	121 (102–140)		113 (90–136)	
PDGFR*β*			0.394		0.087
Negative	80/6/1	164 (154–174)		174 (170–178)	
Positive	116/12/7	163 (153–173)		172 (164–179)	
NG2			0.830		0.216
Negative	93/8/2	145 (136–154)		155 (151–160)	
Positive	103/10/6	165 (155–175)		172 (164–180)	

**Table 6:** Multivariate analysis of disease-free and overall survival in patients with phyllodes tumors.

Included factor	Disease-free survival			Overall survival		
	Hazard ratio	95% CI	*p* value	Hazard ratio	95% CI	*p* value
Histologic grade			**0.002**			N/A
Benign versus borderline/malignant	7.990	2.196–29.07		N/A	N/A	
Stromal cellularity			0.597			0.467
Mild versus moderate/marked	0.625	0.109–3.569		2.862	0.169–48.60	
Stromal atypia			0.886			0.708
Mild versus moderate/marked	0.881	0.158–4.925		0.630	0.056–7.049	
Stromal mitosis			0.854			0.437
0–4/10 HPF versus >4/10 HPF	0.782	0.057–10.81		0.210	0.004–10.70	
Stromal overgrowth			**0.029**			**0.006**
Absent versus present	7.288	1.225–43.35		58.10	3.116–1083	
Tumor margin			0.275			0.187
Circumscribed versus infiltrative	0.468	0.120–1.830		0.321	0.060–1.732	
PDGFR*α* (stromal)			0.867			**0.050**
Negative versus positive	0.879	0.193–4.008		5.486	1.003–30.01	

HPF, high-power field.
